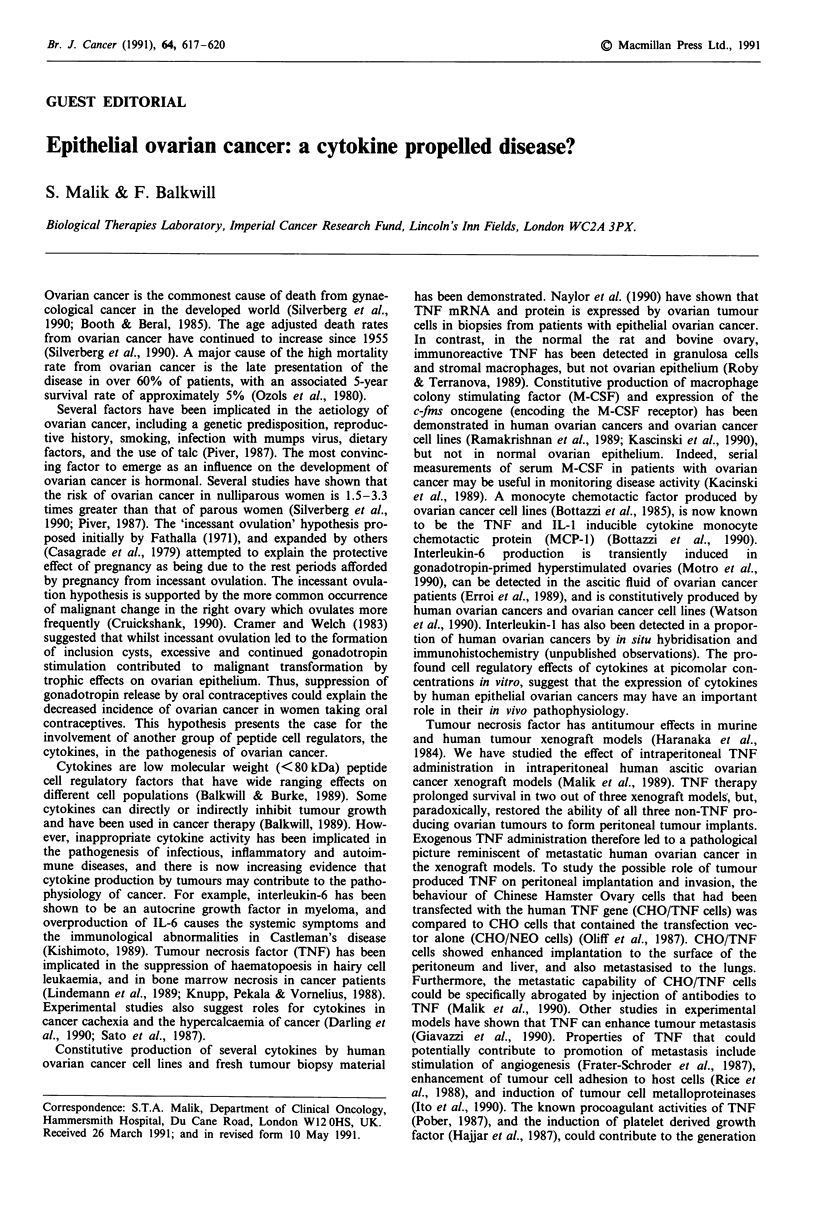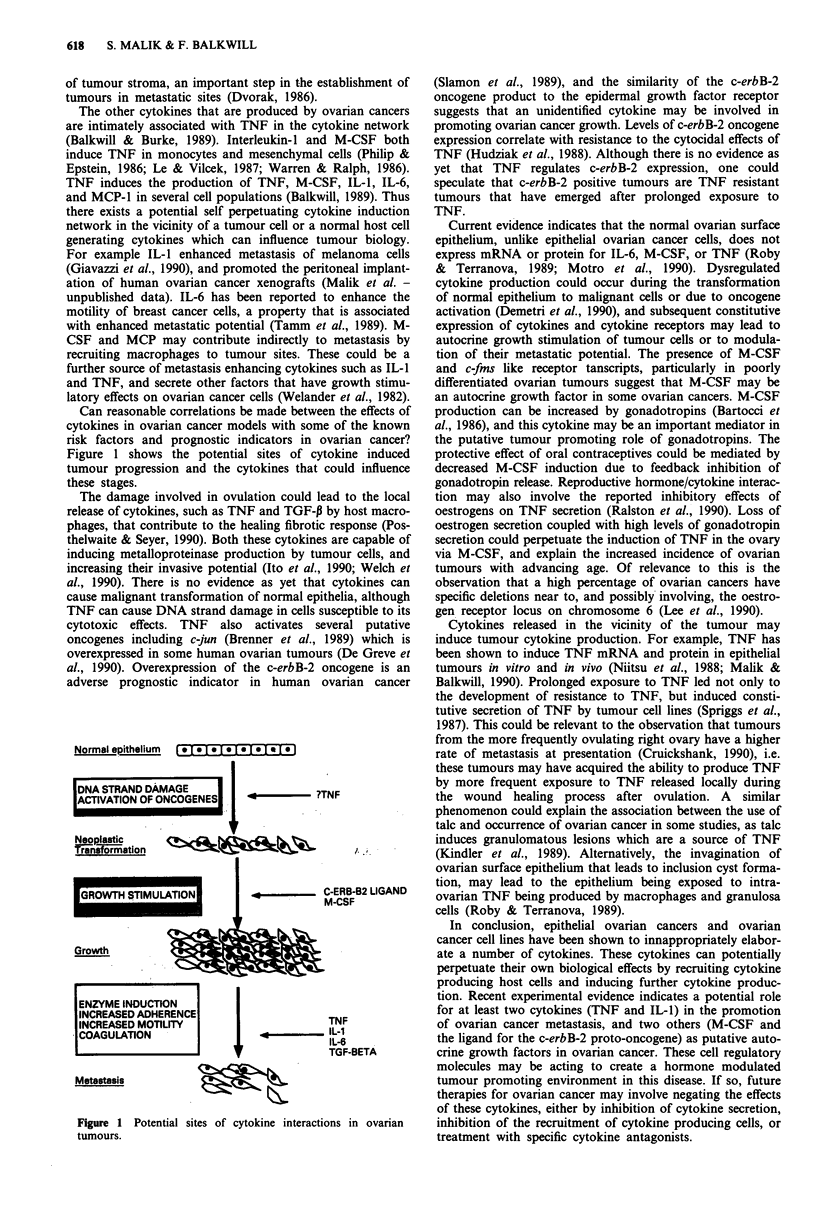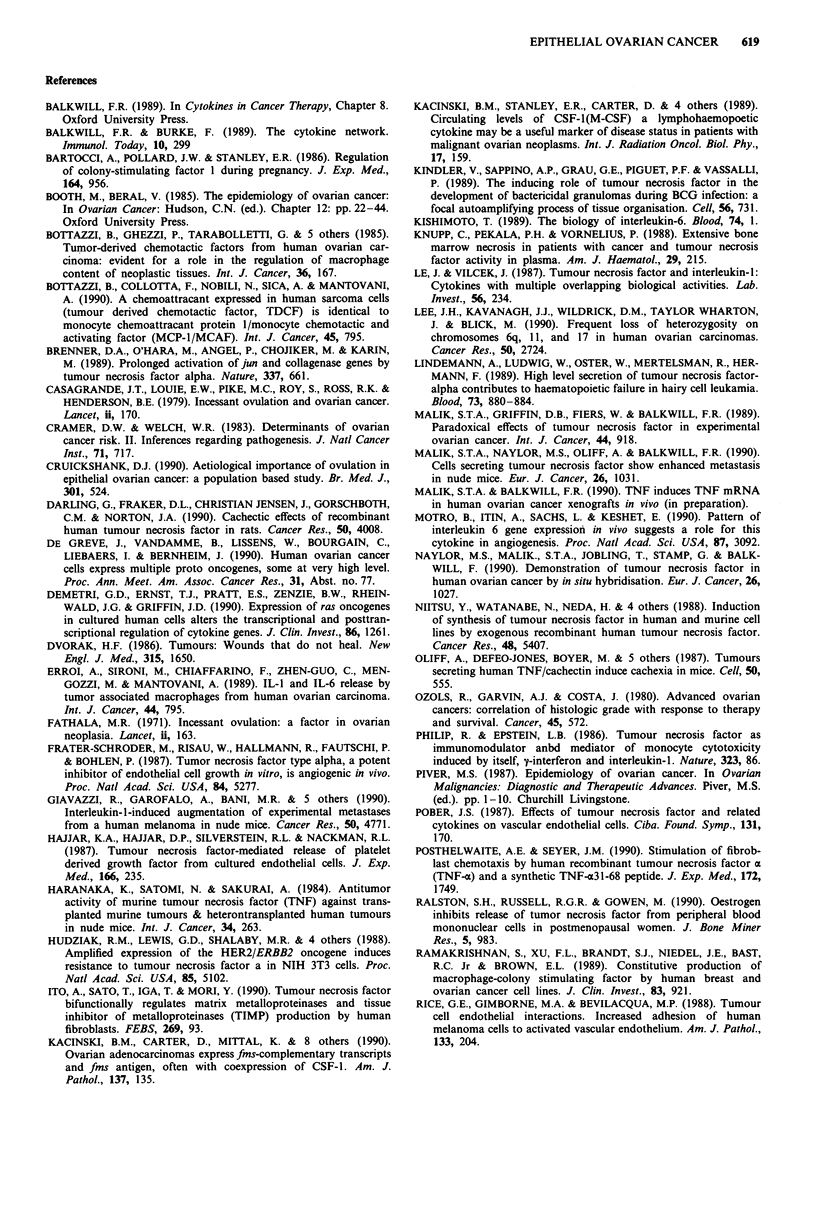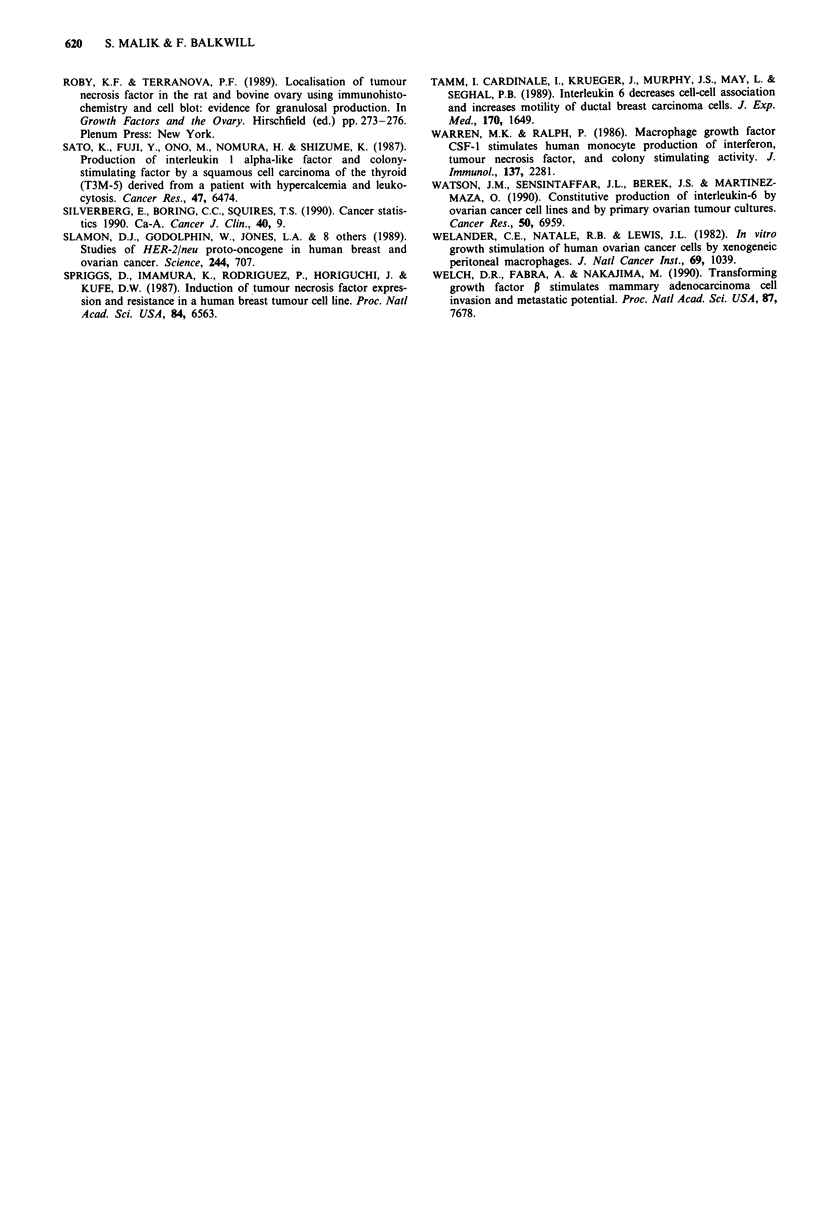# Epithelial ovarian cancer: a cytokine propelled disease?

**DOI:** 10.1038/bjc.1991.372

**Published:** 1991-10

**Authors:** S. Malik, F. Balkwill


Br. J. Cancer (1991), 64, 617-620 © Macmillan Press Ltd., 1991

GUEST EDITORIAL

### Epithelial ovarian cancer: a cytokine propelled disease?

S. Malik & F. Balkwill

*Biological Therapies Laboratory, Imperial Cancer Research Fund, Lincoln's Inn Fields, London WC2A 3PX.*

Ovarian cancer is the commonest cause of death from gynaecological cancer in the developed world (Silverberg *et al.*, 1990; Booth & Beral, 1985). The age adjusted death rates from ovarian cancer have continued to increase since 1955 (Silverberg *et al.*, 1990). A major cause of the high mortality rate from ovarian cancer is the late presentation of the disease in over 60% of patients, with an associated 5-year survival rate of approximately 5% (Ozols *et al.*, 1980).

Several factors have been implicated in the aetiology of ovarian cancer, including a genetic predisposition, reproductive history, smoking, infection with mumps virus, dietary factors, and the use of talc (Piver, 1987). The most convincing factor to emerge as an influence on the development of ovarian cancer is hormonal. Several studies have shown that the risk of ovarian cancer in nulliparous women is 1.5–3.3 times greater than that of parous women (Silverberg *et al.*, 1990; Piver, 1987). The 'incessant ovulation' hypothesis proposed initially by Fathalla (1971), and expanded by others (Casagrade *et al.*, 1979) attempted to explain the protective effect of pregnancy as being due to the rest periods afforded by pregnancy from incessant ovulation. The incessant ovulation hypothesis is supported by the more common occurrence of malignant change in the right ovary which ovulates more frequently (Cruickshank, 1990). Cramer and Welch (1983) suggested that whilst incessant ovulation led to the formation of inclusion cysts, excessive and continued gonadotropin stimulation contributed to malignant transformation by trophic effects on ovarian epithelium. Thus, suppression of gonadotropin release by oral contraceptives could explain the decreased incidence of ovarian cancer in women taking oral contraceptives. This hypothesis presents the case for the involvement of another group of peptide cell regulators, the cytokines, in the pathogenesis of ovarian cancer.

Cytokines are low molecular weight (< 80 kDa) peptide cell regulatory factors that have wide ranging effects on different cell populations (Balkwill & Burke, 1989). Some cytokines can directly or indirectly inhibit tumour growth and have been used in cancer therapy (Balkwill, 1989). However, inappropriate cytokine activity has been implicated in the pathogenesis of infectious, inflammatory and autoimmune diseases, and there is now increasing evidence that cytokine production by tumours may contribute to the pathophysiology of cancer. For example, interleukin-6 has been shown to be an autocrine growth factor in myeloma, and overproduction of IL-6 causes the systemic symptoms and the immunological abnormalities in Castleman's disease (Kishimoto, 1989). Tumour necrosis factor (TNF) has been implicated in the suppression of haematopoesis in hairy cell leukaemia, and in bone marrow necrosis in cancer patients (Lindemann *et al.*, 1989; Knupp, Pekala & Vornelius, 1988). Experimental studies also suggest roles for cytokines in cancer cachexia and the hypercalcaemia of cancer (Darling *et al.*, 1990; Sato *et al.*, 1987).

Constitutive production of several cytokines by human ovarian cancer cell lines and fresh tumour biopsy material has been demonstrated. Naylor *et al.* (1990) have shown that TNF mRNA and protein is expressed by ovarian tumour cells in biopsies from patients with epithelial ovarian cancer. In contrast, in the normal the rat and bovine ovary, immunoreactive TNF has been detected in granulosa cells and stromal macrophages, but not ovarian epithelium (Roby & Terranova, 1989). Constitutive production of macrophage colony stimulating factor (M-CSF) and expression of the *c-fms* oncogene (encoding the M-CSF receptor) has been demonstrated in human ovarian cancers and ovarian cancer cell lines (Ramakrishnan *et al.*, 1989; Kascinski *et al.*, 1990), but not in normal ovarian epithelium. Indeed, serial measurements of serum M-CSF in patients with ovarian cancer may be useful in monitoring disease activity (Kacinski *et al.*, 1989). A monocyte chemotactic factor produced by ovarian cancer cell lines (Bottazzi *et al.*, 1985), is now known to be the TNF and IL-1 inducible cytokine monocyte chemotactic protein (MCP-1) (Bottazzi *et al.*, 1990). Interleukin-6 production is transiently induced in gonadotropin-primed hyperstimulated ovaries (Motro *et al.*, 1990), can be detected in the ascitic fluid of ovarian cancer patients (Erroi *et al.*, 1989), and is constitutively produced by human ovarian cancers and ovarian cancer cell lines (Watson *et al.*, 1990). Interleukin-1 has also been detected in a proportion of human ovarian cancers by *in situ* hybridisation and immunohistochemistry (unpublished observations). The profound cell regulatory effects of cytokines at picomolar concentrations *in vitro*, suggest that the expression of cytokines by human epithelial ovarian cancers may have an important role in their *in vivo* pathophysiology.

Tumour necrosis factor has antitumour effects in murine and human tumour xenograft models (Haranaka *et al.*, 1984). We have studied the effect of intraperitoneal TNF administration in intraperitoneal human ascitic ovarian cancer xenograft models (Malik *et al.*, 1989). TNF therapy prolonged survival in two out of three xenograft models, but, paradoxically, restored the ability of all three non-TNF producing ovarian tumours to form peritoneal tumour implants. Exogenous TNF administration therefore led to a pathological picture reminiscent of metastatic human ovarian cancer in the xenograft models. To study the possible role of tumour produced TNF on peritoneal implantation and invasion, the behaviour of Chinese Hamster Ovary cells that had been transfected with the human TNF gene (CHO/TNF cells) was compared to CHO cells that contained the transfection vector alone (CHO/NEO cells) (Oliff *et al.*, 1987). CHO/TNF cells showed enhanced implantation to the surface of the peritoneum and liver, and also metastasised to the lungs. Furthermore, the metastatic capability of CHO/TNF cells could be specifically abrogated by injection of antibodies to TNF (Malik *et al.*, 1990). Other studies in experimental models have shown that TNF can enhance tumour metastasis (Giavazzi *et al.*, 1990). Properties of TNF that could potentially contribute to promotion of metastasis include stimulation of angiogenesis (Frater-Schroder *et al.*, 1987), enhancement of tumour cell adhesion to host cells (Rice *et al.*, 1988), and induction of tumour cell metalloproteinases (Ito *et al.*, 1990). The known procoagulant activities of TNF (Pober, 1987), and the induction of platelet derived growth factor (Hajjar *et al.*, 1987), could contribute to the generation

Correspondence: S.T.A. Malik, Department of Clinical Oncology, Hammersmith Hospital, Du Cane Road, London W12 0HS, UK.
Received 26 March 1991; and in revised form 10 May 1991.

of tumour stroma, an important step in the establishment of tumours in metastatic sites (Dvorak, 1986).

The other cytokines that are produced by ovarian cancers are intimately associated with TNF in the cytokine network (Balkwill & Burke, 1989). Interleukin-1 and M-CSF both induce TNF in monocytes and mesenchymal cells (Philip & Epstein, 1986; Le & Vilcek, 1987; Warren & Ralph, 1986). TNF induces the production of TNF, M-CSF, IL-1, IL-6, and MCP-1 in several cell populations (Balkwill, 1989). Thus there exists a potential self perpetuating cytokine induction network in the vicinity of a tumour cell or a normal host cell generating cytokines which can influence tumour biology. For example IL-1 enhanced metastasis of melanoma cells (Giavazzi *et al.*, 1990), and promoted the peritoneal implantation of human ovarian cancer xenografts (Malik *et al.* – unpublished data). IL-6 has been reported to enhance the motility of breast cancer cells, a property that is associated with enhanced metastatic potential (Tamm *et al.*, 1989). M-CSF and MCP may contribute indirectly to metastasis by recruiting macrophages to tumour sites. These could be a further source of metastasis enhancing cytokines such as IL-1 and TNF, and secrete other factors that have growth stimulatory effects on ovarian cancer cells (Welander *et al.*, 1982).

Can reasonable correlations be made between the effects of cytokines in ovarian cancer models with some of the known risk factors and prognostic indicators in ovarian cancer? Figure 1 shows the potential sites of cytokine induced tumour progression and the cytokines that could influence these stages.

The damage involved in ovulation could lead to the local release of cytokines, such as TNF and TGF-$\beta$ by host macrophages, that contribute to the healing fibrotic response (Posthelwaite & Seyer, 1990). Both these cytokines are capable of inducing metalloproteinase production by tumour cells, and increasing their invasive potential (Ito *et al.*, 1990; Welch *et al.*, 1990). There is no evidence as yet that cytokines can cause malignant transformation of normal epithelia, although TNF can cause DNA strand damage in cells susceptible to its cytotoxic effects. TNF also activates several putative oncogenes including *c-jun* (Brenner *et al.*, 1989) which is overexpressed in some human ovarian tumours (De Greve *et al.*, 1990). Overexpression of the c-*erb*B-2 oncogene is an adverse prognostic indicator in human ovarian cancer (Slamon *et al.*, 1989), and the similarity of the c-*erb*B-2 oncogene product to the epidermal growth factor receptor suggests that an unidentified cytokine may be involved in promoting ovarian cancer growth. Levels of c-*erb*B-2 oncogene expression correlate with resistance to the cytocidal effects of TNF (Hudziak *et al.*, 1988). Although there is no evidence as yet that TNF regulates c-*erb*B-2 expression, one could speculate that c-*erb*B-2 positive tumours are TNF resistant tumours that have emerged after prolonged exposure to TNF.

**Figure 1** Potential sites of cytokine interactions in ovarian tumours.

Current evidence indicates that the normal ovarian surface epithelium, unlike epithelial ovarian cancer cells, does not express mRNA or protein for IL-6, M-CSF, or TNF (Roby & Terranova, 1989; Motro *et al.*, 1990). Dysregulated cytokine production could occur during the transformation of normal epithelium to malignant cells or due to oncogene activation (Demetri *et al.*, 1990), and subsequent constitutive expression of cytokines and cytokine receptors may lead to autocrine growth stimulation of tumour cells or to modulation of their metastatic potential. The presence of M-CSF and *c-fms* like receptor tanscripts, particularly in poorly differentiated ovarian tumours suggest that M-CSF may be an autocrine growth factor in some ovarian cancers. M-CSF production can be increased by gonadotropins (Bartocci *et al.*, 1986), and this cytokine may be an important mediator in the putative tumour promoting role of gonadotropins. The protective effect of oral contraceptives could be mediated by decreased M-CSF induction due to feedback inhibition of gonadotropin release. Reproductive hormone/cytokine interaction may also involve the reported inhibitory effects of oestrogens on TNF secretion (Ralston *et al.*, 1990). Loss of oestrogen secretion coupled with high levels of gonadotropin secretion could perpetuate the induction of TNF in the ovary via M-CSF, and explain the increased incidence of ovarian tumours with advancing age. Of relevance to this is the observation that a high percentage of ovarian cancers have specific deletions near to, and possibly involving, the oestrogen receptor locus on chromosome 6 (Lee *et al.*, 1990).

Cytokines released in the vicinity of the tumour may induce tumour cytokine production. For example, TNF has been shown to induce TNF mRNA and protein in epithelial tumours *in vitro* and *in vivo* (Niitsu *et al.*, 1988; Malik & Balkwill, 1990). Prolonged exposure to TNF led not only to the development of resistance to TNF, but induced constitutive secretion of TNF by tumour cell lines (Spriggs *et al.*, 1987). This could be relevant to the observation that tumours from the more frequently ovulating right ovary have a higher rate of metastasis at presentation (Cruickshank, 1990), i.e. these tumours may have acquired the ability to produce TNF by more frequent exposure to TNF released locally during the wound healing process after ovulation. A similar phenomenon could explain the association between the use of talc and occurrence of ovarian cancer in some studies, as talc induces granulomatous lesions which are a source of TNF (Kindler *et al.*, 1989). Alternatively, the invagination of ovarian surface epithelium that leads to inclusion cyst formation, may lead to the epithelium being exposed to intra-ovarian TNF being produced by macrophages and granulosa cells (Roby & Terranova, 1989).

In conclusion, epithelial ovarian cancers and ovarian cancer cell lines have been shown to innappropriately elaborate a number of cytokines. These cytokines can potentially perpetuate their own biological effects by recruiting cytokine producing host cells and inducing further cytokine production. Recent experimental evidence indicates a potential role for at least two cytokines (TNF and IL-1) in the promotion of ovarian cancer metastasis, and two others (M-CSF and the ligand for the c-*erb*B-2 proto-oncogene) as putative autocrine growth factors in ovarian cancer. These cell regulatory molecules may be acting to create a hormone modulated tumour promoting environment in this disease. If so, future therapies for ovarian cancer may involve negating the effects of these cytokines, either by inhibition of cytokine secretion, inhibition of the recruitment of cytokine producing cells, or treatment with specific cytokine antagonists.

### References

BALKWILL, F.R. (1989). In *Cytokines in Cancer Therapy*, Chapter 8. Oxford University Press.

BALKWILL, F.R. & BURKE, F. (1989). The cytokine network. *Immunol. Today*, **10**, 299

BARTOCCI, A., POLLARD, J.W. & STANLEY, E.R. (1986). Regulation of colony-stimulating factor 1 during pregnancy. *J. Exp. Med.*, **164**, 956.

BOOTH, M., BERAL, V. (1985). The epidemiology of ovarian cancer: In *Ovarian Cancer*: Hudson, C.N. (ed.). Chapter 12: pp. 22–44. Oxford University Press.

BOTTAZZI, B., GHEZZI, P., TARABOLLETTI, G. & 5 others (1985). Tumor-derived chemotactic factors from human ovarian carcinoma: evident for a role in the regulation of macrophage content of neoplastic tissues. *Int. J. Cancer*, **36**, 167.

BOTTAZZI, B., COLLOTTA, F., NOBILI, N., SICA, A. & MANTOVANI, A. (1990). A chemoattracant expressed in human sarcoma cells (tumour derived chemotactic factor, TDCF) is identical to monocyte chemoattracant protein 1/monocyte chemotactic and activating factor (MCP-1/MCAF). *Int. J. Cancer*, **45**, 795.

BRENNER, D.A., O'HARA, M., ANGEL, P., CHOJIKER, M. & KARIN, M. (1989). Prolonged activation of *jun* and collagenase genes by tumour necrosis factor alpha. *Nature*, **337**, 661.

CASAGRANDE, J.T., LOUIE, E.W., PIKE, M.C., ROY, S., ROSS, R.K. & HENDERSON, B.E. (1979). Incessant ovulation and ovarian cancer. *Lancet*, **ii**, 170.

CRAMER, D.W. & WELCH, W.R. (1983). Determinants of ovarian cancer risk. II. Inferences regarding pathogenesis. *J. Natl Cancer Inst.*, **71**, 717.

CRUICKSHANK, D.J. (1990). Aetiological importance of ovulation in epithelial ovarian cancer: a population based study. *Br. Med. J.*, **301**, 524.

DARLING, G., FRAKER, D.L., CHRISTIAN JENSEN, J., GORSCHBOTH, C.M. & NORTON, J.A. (1990). Cachectic effects of recombinant human tumour necrosis factor in rats. *Cancer Res.*, **50**, 4008.

DE GREVE, J., VANDAMME, B., LISSENS, W., BOURGAIN, C., LIEBAERS, I. & BERNHEIM, J. (1990). Human ovarian cancer cells express multiple proto oncogenes, some at very high level. *Proc. Ann. Meet. Am. Assoc. Cancer Res.*, **31**, Abst. no. 77.

DEMETRI, G.D., ERNST, T.J., PRATT, E.S., ZENZIE, B.W., RHEINWALD, J.G. & GRIFFIN, J.D. (1990). Expression of *ras* oncogenes in cultured human cells alters the transcriptional and posttranscriptional regulation of cytokine genes. *J. Clin. Invest.*, **86**, 1261.

DVORAK, H.F. (1986). Tumours: Wounds that do not heal. *New Engl. J. Med.*, **315**, 1650.

ERROI, A., SIRONI, M., CHIAFFARINO, F., ZHEN-GUO, C., MENGOZZI, M. & MANTOVANI, A. (1989). IL-1 and IL-6 release by tumor associated macrophages from human ovarian carcinoma. *Int. J. Cancer*, **44**, 795.

FATHALA, M.R. (1971). Incessant ovulation: a factor in ovarian neoplasia. *Lancet*, **ii**, 163.

FRATER-SCHRODER, M., RISAU, W., HALLMANN, R., FAUTSCHI, P. & BOHLEN, P. (1987). Tumor necrosis factor type alpha, a potent inhibitor of endothelial cell growth *in vitro*, is angiogenic *in vivo*. *Proc. Natl Acad. Sci. USA*, **84**, 5277.

GIAVAZZI, R., GAROFALO, A., BANI, M.R. & 5 others (1990). Interleukin-1-induced augmentation of experimental metastases from a human melanoma in nude mice. *Cancer Res.*, **50**, 4771.

HAJJAR, K.A., HAJJAR, D.P., SILVERSTEIN, R.L. & NACKMAN, R.L. (1987). Tumour necrosis factor-mediated release of platelet derived growth factor from cultured endothelial cells. *J. Exp. Med.*, **166**, 235.

HARANAKA, K., SATOMI, N. & SAKURAI, A. (1984). Antitumor activity of murine tumour necrosis factor (TNF) against transplanted murine tumours & heterontransplanted human tumours in nude mice. *Int. J. Cancer*, **34**, 263.

HUDZIAK, R.M., LEWIS, G.D., SHALABY, M.R. & 4 others (1988). Amplified expression of the HER2/*ERBB2* oncogene induces resistance to tumour necrosis factor a in NIH 3T3 cells. *Proc. Natl Acad. Sci. USA*, **85**, 5102.

ITO, A., SATO, T., IGA, T. & MORI, Y. (1990). Tumour necrosis factor bifunctionally regulates matrix metalloproteinases and tissue inhibitor of metalloproteinases (TIMP) production by human fibroblasts. *FEBS*, **269**, 93.

KACINSKI, B.M., CARTER, D., MITTAL, K. & 8 others (1990). Ovarian adenocarcinomas express *fms*-complementary transcripts and *fms* antigen, often with coexpression of CSF-1. *Am. J. Pathol.*, **137**, 135.

KACINSKI, B.M., STANLEY, E.R., CARTER, D. & 4 others (1989). Circulating levels of CSF-1(M-CSF) a lymphohaemopoetic cytokine may be a useful marker of disease status in patients with malignant ovarian neoplasms. *Int. J. Radiation Oncol. Biol. Phy.*, **17**, 159.

KINDLER, V., SAPPINO, A.P., GRAU, G.E., PIGUET, P.F. & VASSALLI, P. (1989). The inducing role of tumour necrosis factor in the development of bactericidal granulomas during BCG infection: a focal autoamplifying process of tissue organisation. *Cell*, **56**, 731.

KISHIMOTO, T. (1989). The biology of interleukin-6. *Blood*, **74**, 1.

KNUPP, C., PEKALA, P.H. & VORNELIUS, P. (1988). Extensive bone marrow necrosis in patients with cancer and tumour necrosis factor activity in plasma. *Am. J. Haematol.*, **29**, 215.

LE, J. & VILCEK, J. (1987). Tumour necrosis factor and interleukin-1: Cytokines with multiple overlapping biological activities. *Lab. Invest.*, **56**, 234.

LEE, J.H., KAVANAGH, J.J., WILDRICK, D.M., TAYLOR WHARTON, J. & BLICK, M. (1990). Frequent loss of heterozygosity on chromosomes 6q, 11, and 17 in human ovarian carcinomas. *Cancer Res.*, **50**, 2724.

LINDEMANN, A., LUDWIG, W., OSTER, W., MERTELSMAN, R., HERMANN, F. (1989). High level secretion of tumour necrosis factor-alpha contributes to haematopoietic failure in hairy cell leukamia. *Blood*, **73**, 880–884.

MALIK, S.T.A., GRIFFIN, D.B., FIERS, W. & BALKWILL, F.R. (1989). Paradoxical effects of tumour necrosis factor in experimental ovarian cancer. *Int. J. Cancer*, **44**, 918.

MALIK, S.T.A., NAYLOR, M.S., OLIFF, A. & BALKWILL, F.R. (1990). Cells secreting tumour necrosis factor show enhanced metastasis in nude mice. *Eur. J. Cancer*, **26**, 1031.

MALIK, S.T.A. & BALKWILL, F.R. (1990). TNF induces TNF mRNA in human ovarian cancer xenografts *in vivo* (in preparation).

MOTRO, B., ITIN, A., SACHS, L. & KESHET, E. (1990). Pattern of interleukin 6 gene expression *in vivo* suggests a role for this cytokine in angiogenesis. *Proc. Natl Acad. Sci. USA*, **87**, 3092.

NAYLOR, M.S., MALIK., S.T.A., JOBLING, T., STAMP, G. & BALKWILL, F. (1990). Demonstration of tumour necrosis factor in human ovarian cancer by *in situ* hybridisation. *Eur. J. Cancer*, **26**, 1027.

NIITSU, Y., WATANABE, N., NEDA, H. & 4 others (1988). Induction of synthesis of tumour necrosis factor in human and murine cell lines by exogenous recombinant human tumour necrosis factor. *Cancer Res.*, **48**, 5407.

OLIFF, A., DEFEO-JONES, BOYER, M. & 5 others (1987). Tumours secreting human TNF/cachectin induce cachexia in mice. *Cell*, **50**, 555.

OZOLS, R., GARVIN, A.J. & COSTA, J. (1980). Advanced ovarian cancers: correlation of histologic grade with response to therapy and survival. *Cancer*, **45**, 572.

PHILIP, R. & EPSTEIN, L.B. (1986). Tumour necrosis factor as immunomodulator anbd mediator of monocyte cytotoxicity induced by itself, γ-interferon and interleukin-1. *Nature*, **323**, 86.

PIVER, M.S. (1987). Epidemiology of ovarian cancer. In *Ovarian Malignancies: Diagnostic and Therapeutic Advances*. Piver, M.S. (ed.). pp. 1–10. Churchill Livingstone.

POBER, J.S. (1987). Effects of tumour necrosis factor and related cytokines on vascular endothelial cells. *Ciba. Found. Symp.*, **131**, 170.

POSTHELWAITE, A.E. & SEYER, J.M. (1990). Stimulation of fibroblast chemotaxis by human recombinant tumour necrosis factor α (TNF-α) and a synthetic TNF-α31-68 peptide. *J. Exp. Med.*, **172**, 1749.

RALSTON, S.H., RUSSELL, R.G.R. & GOWEN, M. (1990). Oestrogen inhibits release of tumor necrosis factor from peripheral blood mononuclear cells in postmenopausal women. *J. Bone Miner Res.*, **5**, 983.

RAMAKRISHNAN, S., XU, F.L., BRANDT, S.J., NIEDEL, J.E., BAST, R.C. Jr & BROWN, E.L. (1989). Constitutive production of macrophage-colony stimulating factor by human breast and ovarian cancer cell lines. *J. Clin. Invest.*, **83**, 921.

RICE, G.E., GIMBORNE, M.A. & BEVILACQUA, M.P. (1988). Tumour cell endothelial interactions. Increased adhesion of human melanoma cells to activated vascular endothelium. *Am. J. Pathol.*, **133**, 204.

ROBY, K.F. & TERRANOVA, P.F. (1989). Localisation of tumour necrosis factor in the rat and bovine ovary using immunohistochemistry and cell blot: evidence for granulosal production. In *Growth Factors and the Ovary*. Hirschfield (ed.) pp. 273–276. Plenum Press: New York.

SATO, K., FUJI, Y., ONO, M., NOMURA, H. & SHIZUME, K. (1987). Production of interleukin 1 alpha-like factor and colony-stimulating factor by a squamous cell carcinoma of the thyroid (T3M-5) derived from a patient with hypercalcemia and leukocytosis. *Cancer Res.*, **47**, 6474.

SILVERBERG, E., BORING, C.C., SQUIRES, T.S. (1990). Cancer statistics 1990. Ca-A. *Cancer J. Clin.*, **40**, 9.

SLAMON, D.J., GODOLPHIN, W., JONES, L.A. & 8 others (1989). Studies of *HER-2/neu* proto-oncogene in human breast and ovarian cancer. *Science*, **244**, 707.

SPRIGGS, D., IMAMURA, K., RODRIGUEZ, P., HORIGUCHI, J. & KUFE, D.W. (1987). Induction of tumour necrosis factor expression and resistance in a human breast tumour cell line. *Proc. Natl Acad. Sci. USA*, **84**, 6563.

TAMM, I. CARDINALE, I., KRUEGER, J., MURPHY, J.S., MAY, L. & SEGHAL, P.B. (1989). Interleukin 6 decreases cell-cell association and increases motility of ductal breast carcinoma cells. *J. Exp. Med.*, **170**, 1649.

WARREN, M.K. & RALPH, P. (1986). Macrophage growth factor CSF-1 stimulates human monocyte production of interferon, tumour necrosis factor, and colony stimulating activity. *J. Immunol.*, **137**, 2281.

WATSON, J.M., SENSINTAFFAR, J.L., BEREK, J.S. & MARTINEZ-MAZA, O. (1990). Constitutive production of interleukin-6 by ovarian cancer cell lines and by primary ovarian tumour cultures. *Cancer Res.*, **50**, 6959.

WELANDER, C.E., NATALE, R.B. & LEWIS, J.L. (1982). *In vitro* growth stimulation of human ovarian cancer cells by xenogeneic peritoneal macrophages. *J. Natl Cancer Inst.*, **69**, 1039.

WELCH, D.R., FABRA, A. & NAKAJIMA, M. (1990). Transforming growth factor $\beta$ stimulates mammary adenocarcinoma cell invasion and metastatic potential. *Proc. Natl Acad. Sci. USA*, **87**, 7678.